# Development of Functional Bread Fortified with Hawthorn Leaf Phenolic Extract: Evaluation of Technological Performance and Phenolic Bioaccessibility

**DOI:** 10.1002/fsn3.72217

**Published:** 2026-08-04

**Authors:** Semra Topuz Türker, Esra Esin, Mustafa Bayram, Cemal Kaya

**Affiliations:** ^1^ Department of Food Engineering, Faculty of Engineering and Architecture Tokat Gaziosmanpaşa University Tokat Turkey

**Keywords:** bread fortification, functional food, hawthorn leaf phenolic extract, in vitro digestion, phenolic bioaccessibility

## Abstract

The leaves of hawthorn trees (*Crataegus* spp.) are rich in phenolic compounds that have well‐documented antioxidant properties. However, the use of these compounds in bakery products and their behavior during gastrointestinal digestion remain largely unexplored. This study investigated the effects of incorporating different concentrations (0.30%–1.50%) of hawthorn leaf phenolic extract (HLPE) into bread formulations on its technological properties, total phenolic content (TPC) and total flavonoid content (TFC), antioxidant capacity, and in vitro gastrointestinal bioaccessibility. Bread supplementation with HLPE significantly increased the TPC from 64.77 to 272.84 mg GAE/100 g and the TFC from 50.69 to 234.23 mg QE/100 g, markedly enhancing antioxidant activity. ABTS, DPPH, and FRAP values reached 308.63, 162.47, and 193.47 mg TE/100 g, respectively, at 1.50% HLPE supplementation. However, increasing HLPE levels adversely affected loaf volume, specific volume, texture, and color, resulting in a pronounced quality deterioration at the highest supplementation level. Simulated gastrointestinal digestion revealed that post gastric fraction (PG) increased phenolic extractability (126.31–297.06 mg GAE/100 g), whereas dialyzable fraction (IN) showed lower phenolic recovery (66.48–84.49 mg GAE/100 g), corresponding to bioaccessibility values of approximately 13.17%–14.03%. The highest TPC and TFC values were observed in the non‐dialyzable fraction (OUT), which retained high antioxidant activity. Among all formulations, 1.20% HLPE was identified as the optimal level, balancing enhanced bioactive properties with acceptable technological quality. These findings suggest that HLPE‐enriched bread could serve as a promising functional food, providing phenolic compounds throughout the digestive process.

## Graphical abstract

This study investigates the production of hawthorn ( *Crataegus azarolus* ) leaf extract–fortified functional bread. Ultrasound‐assisted extraction was optimized using Response Surface Methodology (Box–Behnken design) by adjusting extraction time, temperature, and ethanol concentration. The optimized process produced a high‐quality phenolic extract, which was freeze‐dried and incorporated into bread formulations at different supplementation levels. HLPE enrichment significantly improved the functional properties of bread by increasing TPC, TFC, and antioxidant capacity (ABTS, DPPH, and FRAP assays). However, higher HLPE levels negatively affected technological quality, including loaf volume, specific volume, texture, and crust/crumb color, indicating a balance between functionality and structural quality. In vitro gastrointestinal digestion (INFOGEST model) showed progressive release of phenolic compounds during gastric conditions, while intestinal dialyzable fractions exhibited limited bioaccessibility. Most phenolics remained in the non‐dialyzable fraction, suggesting strong matrix interactions and potential colonic delivery. Overall, an intermediate HLPE level provided the best balance between functional enhancement and bread quality, highlighting hawthorn leaf phenolics as promising ingredients for antioxidant‐enriched bakery products.

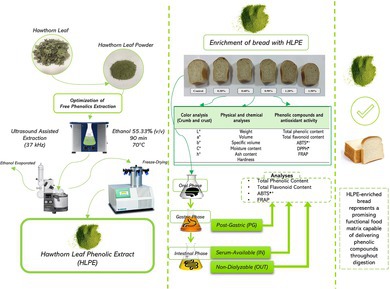

## 1 Introduction

Bread is a cereal‐based food product that is widely consumed around the world and recognized as an important part of many societies' diets (Falsafi et al. 2025). The conventional bread‐making process consists of combining cereal flour with water, salt, and yeast to obtain a viscoelastic dough, which is later fermented and baked in a controlled manner (Baiano et al. 2015). In addition to contributing substantially to daily energy intake, bread plays a pivotal role in a balanced diet due to its carbohydrate, protein, dietary fiber, vitamin, and mineral content (Purkiewicz et al. 2024). Nevertheless, the widespread use of refined flours in modern bread production significantly reduces the levels of dietary fiber and bioactive compounds, which could potentially reduce the functional and nutraceutical value of the final product (Czajkowska‐González et al. 2021). Consequently, recent studies have increasingly emphasized that bread should be considered not only as a source of essential nutrients, but also as a functional food with potential health‐promoting properties (Rahaie et al. 2014; Amoah et al. 2022).

Functional foods are conceptualized as food products that, in addition to supplying essential nutrients, contain bioactive constituents that trigger favorable physiological responses and may reduce the risk of chronic diseases (Fekete et al. 2025; Frumuzachi et al. 2025). From a public health perspective, fortifying widely consumed staple foods, such as bread, with functional components is considered a strategic approach. The literature reports that incorporating extracts derived from grape seeds, green tea, olive leaves, pomegranate peels and various medicinal and aromatic plants into bread formulations enhances the final product's antioxidant activity and phenolic content, as well as improving its functional properties (Altunkaya et al. 2013; Meral and Doǧan 2013; Baiano et al. 2015; Alkay et al. 2022). Furthermore, the influence of these additions on technological attributes varies, affecting parameters such as dough rheology, bread volume, crumb microstructure, color and texture (Meral and Doǧan 2013; Zarzycki et al. 2024).

Hawthorn (*Crataegus* spp.) is a plant species that is particularly rich in phenolic compounds. It is widely used in traditional and complementary medicine (Cui et al. 2024). The leaves of the hawthorn tree are distinguished by their high concentrations of bioactive constituents, particularly the flavonoids hyperoside, vitexin and rutin, as well as the phenolic acids and oligomeric procyanidins (Shu et al. 2023; Bai et al. 2024). Extensive research has shown that these compounds possess strong antioxidant activity and are effective in scavenging free radicals. They also provide multiple health‐promoting effects, particularly with regard to cardiovascular function (Dikici and Köksal 2023; Kępińska‐Pacelik and Biel 2026). Consequently, hawthorn leaves and their extracts have attracted significant attention as functional ingredients with the potential to be used in the development of foods that enhance health and nutraceutical formulations (Zhang et al. 2022). However, there is currently a lack of research explicitly examining the use of hawthorn leaf extracts in bread formulations.

The functional efficacy of phenolic compounds in food systems is determined not only by their total concentration but also by their release and bioaccessibility during gastrointestinal digestion (Shahidi and Peng 2018). This concept is known as “bioaccessibility” and refers to the fraction of phenolic compounds released in a soluble form that becomes available for absorption following digestion (Kalita et al. 2025). The bioaccessibility of phenolic compounds in complex food matrices, such as bread, which are rich in starch and protein, can be influenced by numerous factors. These include phenolic–protein and phenolic–starch interactions, baking processes and extract concentration (Angelino et al. 2017). Therefore, in breads enriched with functional components, the assessment of both total phenolic content and bioaccessibility under in vitro digestion conditions is crucial (Angelino et al. 2017; Świeca et al. 2017).

The present study aims to investigate the effects of incorporating hawthorn leaf phenolic extracts into bread at different concentrations on the technological and bioactive properties of the final product. Additionally, this study will assess the bioaccessibility of phenolic compounds through simulated in vitro gastrointestinal digestion. While previous studies have reported improvements in the nutritional and antioxidant properties of bread fortified with various plant extracts (Tolve et al. 2021; Costa et al. 2025; Zahorec et al. 2025), these studies have primarily examined technological, sensory, and compositional characteristics rather than the gastrointestinal fate of bioactive compounds. However, limited information is available regarding the bioaccessibility of phenolic compounds in plant‐extract‐fortified bread systems following in vitro digestion (Bashmil et al. 2025; Nicolau et al. 2026). To the best of our knowledge, no studies have examined bread fortified with hawthorn leaf extracts with respect to the bioaccessibility of phenolic compounds. Therefore, this study addresses a gap in the literature and provides novel insights into the potential of hawthorn‐enriched bread as a functional food.

## 2 Material and Methods

### 2.1 Materials

The leaves of the hawthorn ( *Crataegus azarolus* ) were harvested in September 2022 from the Soğukpınar area of Çayır Village in the Zile district of the Tokat Province in Türkiye, at an altitude of 1860 m above sea level. Bread wheat flour ( *Triticum aestivum* L.; Mis Un, Türkiye), baker's yeast ( *Saccharomyces cerevisiae* ; Yuva Maya, Türkiye), table salt (NaCl; Saray Tuz, Türkiye), and granulated sugar (sucrose; Tatküpü, Türkiye) were obtained from a local market in Tokat, Türkiye, in December 2024.

### 2.2 Extraction and Optimization of Phenolic Compounds from Hawthorn Leaves

The free phenolic compounds from hawthorn leaves were extracted under previously optimized conditions (Esin et al. 2026). Briefly, 400 mg of leaf powder was mixed with 10 mL of extraction solvent and subjected to ultrasound‐assisted extraction in a bath operating at 37 kHz in continuous mode. The extraction conditions were optimized through Response Surface Methodology (RSM) employing a Box–Behnken experimental design, considering extraction time (X_1_: 5–90 min), temperature (X_2_: 30°C–70°C), and ethanol concentration (X_3_: 10%–90% v/v ethanol in aqueous solution) as independent variables.

Following extraction, the mixtures were centrifuged at 6000 rpm for 5 min, and the supernatants were separated by filtration through coarse‐grade filter paper. The resulting filtrates were collected and used for subsequent analyses. The optimization procedure was conducted using total phenolic content (TPC) and total flavonoid content (TFC) as the selected response parameters. The fitted quadratic models for both responses demonstrated statistical significance (*p* < 0.01), and the lack‐of‐fit analysis indicated non‐significant results at the 95% confidence level (*p* > 0.05). For TPC, the model exhibited excellent fit and predictive ability (*R* ^2^ = 0.99, Adj‐*R* ^2^ = 0.99, Adequate precision = 47.23, PRESS = 111.28, C.V.% = 2.70). Similarly, the model for TFC also demonstrated high reliability (*R* ^2^ = 0.99, Adj‐*R* ^2^ = 0.98, Adequate precision = 32.40, PRESS = 111.02, C.V.% = 3.68). Using a desirability function approach, the optimal extraction conditions were determined to be 90 min, 70°C, and 55.33% ethanol solvent, yielding predicted TPC and TFC values of 78.61 mg GAE/g DW and 63.95 mg QE/g DW, respectively. To verify the optimized model, experiments were carried out in triplicate, yielding TPC and TFC values of 79.77 mg GAE/g DW and 64.15 mg QE/g DW, respectively. Scale‐up extraction was performed under these optimized UAE conditions. Ethanol was removed from the extract using a rotary evaporator, and the remaining aqueous solution was subsequently freeze‐dried. For lyophilization, approximately 250 mL extract was placed into each 500 mL round‐bottom flask, with a freeze dryer equipped with eight flask adapters. The freeze‐drying process was conducted at approximately −50°C under a vacuum of ~1 mbar. The resulting lyophilized hawthorn leaf phenolic extract (HLPE) was then used in the bread‐making process.

### 2.3 Production of Bread Fortified with Hawthorn Leaf Phenolic Extract

Bread was produced based on 100 g of flour, using 2.20% yeast, 2.00% salt, 1.00% sugar, and 56.00% water. The levels of HLPE added to the bread formulations were determined through preliminary trials and selected at 0.3%, 0.6%, 0.9%, 1.2%, and 1.5% (w/w on a flour basis), considering that lower concentrations may result in insufficient functional effectiveness, whereas higher levels could potentially induce undesirable technological effects on dough handling and bread quality. Initially, the yeast, sugar, salt, water, and HLPE were mixed and kneaded together to form a homogeneous, well‐developed dough. After kneading, the dough underwent bulk fermentation at 35°C ± 1°C and 75% relative humidity for 60 min.

**FIGURE 1:**
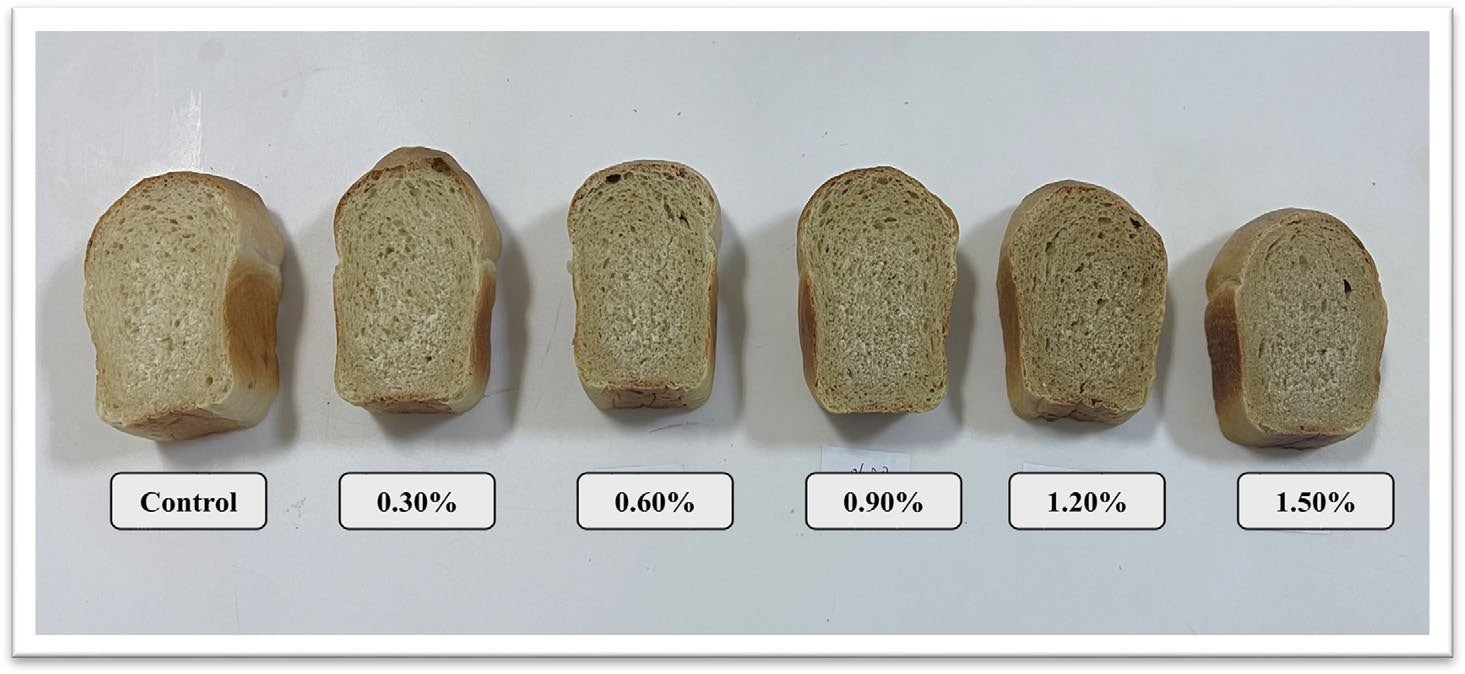
Appearance of bread samples fortified with different concentrations of hawthorn leaf phenolic extract.

Following bulk fermentation, the excess gas was expelled. The dough was then divided into 150 g portions, placed in baking pans and left to undergo final proofing at 43°C ± 1°C with a relative humidity of 75% for 30 min. The dough pieces were then baked at 220°C for 16 min. Following the baking process, the bread was cooled to room temperature for 5 h prior to physicochemical and bioactive analyses (Şen 2013; Cingöz et al. 2022). Figure 1 shows images of the resulting bread samples corresponding to the different HLPE concentrations.

### 2.4 Physical Analyses

The weight of each loaf was measured using a digital balance after cooling. Loaf volume was determined using the rapeseed displacement method, which is based on the principle of volume displacement in a container of known dimensions. The specific loaf volume was calculated as the ratio of bread volume to bread weight (Elgün et al. 2005).

### 2.5 Total Dry Matter and Ash Content Analysis

The total dry matter content of bread samples was determined gravimetrically by drying approximately 3.0 ± 0.5 g of sample in a drying oven at 105°C ± 2°C until a constant weight was reached. Prior to analysis, drying dishes were brought to constant weight. Ash content was determined by incinerating approximately 1.0 ± 0.2 g of bread sample in porcelain crucibles in a muffle furnace at 600°C ± 10°C until white ash was obtained, following preconditioning of the crucibles to constant weight. Results of total dry matter and total ash content were expressed as g/100 g bread (%), based on AOAC standard analytical methods (AOAC 2005).

### 2.6 Instrumental Color Analysis

The color of the samples was measured using a colorimeter operating within the International Commission on Illumination (CIE) color space. The color coordinates L* (lightness), a* (red–green axis), and b* (yellow–blue axis) were determined for each sample. Based on these parameters, chroma (C*) and hue angle (h°) values were calculated (CIE 1976).

### 2.7 Instrumental Texture Analysis

The hardness of the bread samples was determined using a texture analyzer equipped with a stainless‐steel probe. The maximum force applied during compression was recorded and expressed in Newtons (N) (Cingöz 2018). The operating conditions of the texture analyzer were set as follows: Test speed (V_test_) 35 mm/min, return speed (V_return_) 500 mm/min, pre‐test speed (V_pos1_) 500 mm/min, post‐test speed (V_pos2_) 10 mm/min, maximum deformation distance (L_max_) 10 mm, and trigger force (F_v_) 0.1 N.

### 2.8 Extraction of Bread Samples

Bread samples were extracted using a solvent extraction method. The extraction conditions were determined based on preliminary experiments to ensure efficient recovery of phenolic compounds. Extraction was performed using an acidified hydroethanolic solution (50% v/v ethanol–water with 0.1% HCl). Briefly, 5 g of bread sample was mixed with 25 mL of the solvent mixture and subjected to extraction in a shaking water bath at 40°C for 120 min under continuous agitation. Centrifugation at 6000 rpm for 10 min was applied after extraction to separate the solid material from the liquid phase. The supernatant was recovered and filtered to ensure the removal of any remaining solid matter. These extracts were further analyzed to evaluate TPC, TFC, and antioxidant capacity.

### 2.9 Determination of TPC

TPC values of the samples were determined using the Folin–Ciocalteu (FC) method. In brief, 100 μL of the extract was mixed with 200 μL of the FC reagent and 2 mL of distilled water, and the mixture was allowed to stand at room temperature for 3 min. The assay mixture was prepared by adding 1 mL of 20% (w/v) Na_2_CO_3_ solution, followed by vortexing and incubation at room temperature for 1 h, and the absorbance was finally measured at 765 nm using a spectrophotometer. TPC values were calculated from a gallic acid calibration curve and expressed as mg gallic acid equivalents (GAE)/100 g of bread, accounting for dilution factors (Topuz and Bayram 2022).

### 2.10 Determination of TFC

TFC values of the samples were determined using a modified aluminum chloride colorimetric method according to Gaafar and Salama (2013). In brief, 500 μL of the extract was mixed with 2 mL of distilled water and 150 μL of 5% NaNO_2_ solution. After 5 min at room temperature, 150 μL of 10% AlCl_3_ solution was added, followed by incubation for an additional 5 min. The reaction mixture was supplemented with 1 mL of 1 M NaOH and 1.2 mL of distilled water, vortexed, and then analyzed at 415 nm. TFC was calculated from a quercetin calibration curve and reported as mg quercetin equivalents (QE)/100 g of bread.

### 2.11 Determination of Antioxidant Activities

The ABTS cation radical scavenging activity of the samples was determined using the spectrophotometric method developed by Re et al. (1999). The DPPH free radical scavenging activity was assessed according to Blasi et al. (2016), while ferric reducing antioxidant power (FRAP) of the samples was measured following the method described by Benzie and Strain (1996). Calibration curves were constructed using Trolox as the standard, and the results were expressed as mg Trolox equivalent (TE)/100 g of bread.

### 2.12 In Vitro Gastrointestinal Digestion and Bioaccessibility Evaluation

In vitro bioaccessibility was assessed using a simulated gastrointestinal digestion model based on the INFOGEST protocol (Minekus et al. 2014), combined with a dialysis approach during the intestinal phase as described by McDougall et al. (2005). Simulated salivary fluid (SSF), simulated gastric fluid (SGF), and simulated intestinal fluid (SIF) were prepared to mimic physiological and chemical conditions of the human gastrointestinal tract. SSF contained KCl (15.1 mmol/L), KH_2_PO_4_ (3.7 mmol/L), NaHCO_3_ (13.6 mmol/L), MgCl_2_.6H_2_O (0.15 mmol/L), and (NH_4_)_2_CO_3_ (0.06 mmol/L). SGF consisted of KCl (6.9 mmol/L), KH_2_PO_4_ (0.9 mmol/L), NaHCO_3_ (25 mmol/L), NaCl (47.2 mmol/L), MgCl_2_.6H_2_O (0.1 mmol/L), and (NH_4_)_2_CO_3_ (0.5 mmol/L). SIF was composed of KCl (6.8 mmol/L), KH_2_PO_4_ (0.8 mmol/L), NaHCO_3_ (85 mmol/L), NaCl (38.4 mmol/L), and MgCl_2_·6H_2_O (0.33 mmol/L). A 0.3 mol/L CaCl_2_.2H_2_O solution was freshly prepared and used to ensure enzymatic activation. Enzyme activities were determined prior to digestion, and enzyme concentrations were adjusted accordingly. Pepsin activity was measured using bovine hemoglobin, while the trypsin activity of pancreatin was evaluated using *p*‐toluene‐sulfonyl‐L‐arginine methyl ester (TAME). All digestive fluids were equilibrated at 37°C before use.

Digestion was performed sequentially under simulated oral, gastric, and intestinal conditions. Briefly, samples (5 g) were mixed with SSF containing α‐amylase (final activity 75 U/mL), 25 μL of 0.3 M CaCl_2_(H_2_O)_2_, and 975 μL of distilled water and incubated at 37°C for 2 min with shaking (100 rpm).

For the gastric phase, the oral bolus (10 mL) was mixed with 7.5 mL of SGF, 1.6 mL of pepsin solution (final activity: 2000 U/mL), and 5 μL of 0.3 M CaCl_2_.2H_2_O. The mixture was then acidified with 0.2 mL of 1 M HCl and adjusted with 695 μL of distilled water. The system was incubated in a shaking incubator at 37°C and 100 rpm for 2 h to simulate gastric digestion. After completion of this phase, 2 mL of the gastric digest (post gastric fraction, PG) was collected for analysis. Prior to intestinal digestion, the remaining gastric chyme was adjusted to a final volume of 20 mL by adding 2 mL of distilled water to ensure consistency of the subsequent digestion stage.

For the intestinal phase, the gastric chyme (20 mL) was combined with 11 mL of SIF, 5 mL of pancreatin solution (final activity: 100 U/mL), 2.5 mL of bile salt solution (160 mM), 40 μL of 0.3 M CaCl_2_.2H_2_O, 150 μL of NaOH, and 1.31 mL of distilled water. A dialysis membrane (14 kDa molecular weight cut‐off) was filled with 2 M NaHCO_3_ solution, securely sealed, and introduced into the intestinal digestion system to simulate the absorption process. The system was then incubated at 37°C and 75 rpm for 2 h in a shaking incubator to complete intestinal digestion. At the end of digestion, samples from inside (IN) and outside (OUT) the dialysis membrane were collected. The dialysis step was used to simulate intestinal absorption and distinguish between bioaccessible (dialyzable) and non‐bioaccessible (non‐dialyzable) fractions. However, it should be noted that this in vitro model does not fully capture the complexity of human intestinal transport processes. This is an inherent limitation of the method. Enzymatic activity was terminated using Pefabloc (500 mM), and the digesta were centrifuged (6000 rpm, 15 min, 4°C). The supernatants were stored at −86°C until analysis.

Samples were collected at three critical stages of the digestion process:

- PG (Post gastric fraction): Representing the sample exiting the stomach,
- IN (Dialyzable fraction): Dialyzable compounds that can potentially be absorbed into the bloodstream,
- OUT (Non‐dialyzable fraction): Compounds that are not absorbed in the intestine.

All in vitro gastrointestinal digestion experiments were performed in duplicate. The bioaccessibility of TPC, TFC, and antioxidant capacity (ABTS and FRAP) was calculated as the proportion of the dialyzable intestinal fraction (IN) relative to the total amount recovered after the intestinal digestion step (IN + OUT), according to the following equation:

$$\text{Bioaccessibility}\;\left(\%\right)=\left[\left(\text{IN fraction}\right)/\left(\text{IN fraction}+\text{OUT fraction}\right)\right]\times 100$$

### 2.13 Statistical Analysis

Data were analyzed using SPSS software (version 22.0; IBM, USA). Prior to statistical analysis, the assumptions of normality and homogeneity of variances were evaluated using the Shapiro–Wilk and Levene tests, respectively. Since all variables satisfied these assumptions (*p* > 0.05), significant differences among treatment means were assessed using one‐way analysis of variance (ANOVA) followed by Duncan's multiple range test at *p* < 0.05. Pearson correlation coefficients (*r*) were also calculated. Bread production was performed in two independent batches, and each batch was analyzed in triplicate to ensure reproducibility of the results. The in vitro gastrointestinal digestion was independently performed in duplicate, and all analytical determinations were conducted in triplicate for each digestion replicate.

## 3 Results and Discussion

### 3.1 Physical and Chemical Characteristics of Bread Samples

The results of the physical and chemical analyses (weight, volume, specific volume, moisture content, ash content, and hardness) of the control bread and HLPE‐fortified bread samples at different concentrations (0.30%, 0.60%, 0.90%, 1.20%, and 1.50%) are presented in Table 1.

**TABLE 1:** Physical and chemical properties of control bread and HLPE‐fortified bread samples at different concentrations (0.30%–1.50%).

Bread samples	Weight (g)	Volume (cm^3^)	Specific volume (cm^3^/g)	Moisture content (%)	Ash content (%)	Hardness (N)
Control	116.75 ± 0.57^d^ *	357.00 ± 24.25^a^	3.06 ± 0.21^a^	25.68 ± 0.22^b^	2.05 ± 0.01^a^	6.21 ± 0.20^f^
0.30% HLPE	118.39 ± 0.89^b^	325.50 ± 21.00^b^	2.75 ± 0.19^b^	25.85 ± 0.28^b^	2.03 ± 0.01^a^	7.54 ± 0.39^e^
0.60% HLPE	117.23 ± 0.45^cd^	315.00 ± 17.15^b^	2.69 ± 0.14^b^	25.74 ± 0.12^b^	2.02 ± 0.02^a^	8.48 ± 0.40^d^
0.90% HLPE	117.65 ± 0.30^bcd^	309.75 ± 10.50^b^	2.63 ± 0.08^b^	24.97 ± 0.12^c^	2.00 ± 0.04^a^	9.25 ± 0.48^c^
1.20% HLPE	121.10 ± 0.92^a^	304.50 ± 12.12^b^	2.51 ± 0.11^b^	26.64 ± 0.51^a^	1.96 ± 0.09^a^	10.81 ± 0.55^b^
1.50% HLPE	117.81 ± 0.48^bc^	257.25 ± 20.11^c^	2.18 ± 0.17^c^	25.69 ± 0.21^b^	1.87 ± 0.18^a^	11.90 ± 0.65^a^

*Note:* Results are given as mean ± standard deviation.

Abbreviation: HLPE, Hawthorn leaf phenolic extract.

* Small letters in the same column show difference between bread samples (*p* < 0.05).

The weight of the bread samples varied between 116.75 g (control group) and 121.10 g (1.20% HLPE), and the addition of HLPE had a statistically significant effect on bread weight (*p* < 0.05). An upward trend was observed as the HLPE concentration increased up to 1.20%. However, a decrease was recorded at 1.50%. These variations in bread weight may be attributed to the influence of HLPE on the water‐holding capacity of the dough. Phenolic compounds are known to interact with flour proteins (particularly gluten) and carbohydrates through non‐covalent interactions such as hydrogen bonding, which can modify the dough matrix and enhance water retention (Girard and Awika 2020; Schefer et al. 2021). As a result, reduced water evaporation during baking may contribute to the increased final product weight. The increase observed up to the 1.20% ratio might have supported the water‐binding properties of the extract; however, the decline at the 1.50% level could be explained by high concentrations of polyphenols weakening the gluten network, thereby adversely affecting gas retention and moisture preservation capacity.

The volume of the bread samples was found to range between 257.25 cm^3^ and 357.00 cm^3^. The addition of HLPE caused a statistically significant decrease in bread volume (*p* < 0.05). While the highest volume value was measured in the control group (357.00 cm^3^), a gradual decline in volume values was recorded as the HLPE concentration increased. All HLPE‐added groups between 0.30% and 1.20% were found to be statistically similar (*p* > 0.05), whereas the lowest volume value was identified with a distinct difference in samples containing 1.50% HLPE (257.25 cm^3^). This reduction in volume can be linked to the interactions between phenolic compounds and gluten proteins, particularly gliadin and glutenin. Phenolic compounds in hawthorn leaves may form protein–polyphenol complexes and influence the gluten network through changes in disulfide bonding and non‐covalent interactions, weakening gluten elasticity and reducing its gas‐holding capacity, which in turn can decrease bread loaf volume. Additionally, it is likely that high concentrations of phenolics exerted an inhibitory effect on yeast ( *Saccharomyces cerevisiae* ) activity, limiting gas production during fermentation (Xu et al. 2019). The sharp decline at the 1.50% dosage suggests that the dough matrix significantly lost its structural integrity under the high phenolic load. Similar findings have been reported in the literature. In a study conducted by Şen (2013), wheat flour was supplemented with grape seed, pomegranate seed, and rosehip seed flours at different levels (5.00%, 7.50%, and 10.00%). The results indicated that increasing the proportion of seed flours in the dough formulation led to a reduction in bread loaf volume. In another study, defatted blackcurrant and strawberry seed flours were incorporated at levels of 5.00%, 10.00%, and 15.00% in gluten‐free bread production. The addition of blackcurrant seed flour resulted in a decrease in bread volume, whereas strawberry seed flour was reported to increase loaf volume (Korus et al. 2012).

The specific volume values of the bread samples ranged from 2.18 cm^3^/g to 3.06 cm^3^/g, with HLPE addition causing a statistically significant decrease (*p* < 0.05). The control sample exhibited the highest specific volume. A decreasing trend was observed with the addition of HLPE; while the differences between samples containing 0.30% to 1.20% HLPE were found to be statistically insignificant (*p* > 0.05), the lowest specific volume was recorded in the sample containing 1.50% HLPE. Specific volume is one of the most critical parameters reflecting bread quality and the porosity of the internal structure (Monteiro et al. 2021). The decrease in specific volume can be explained by the weakening of the dough's gas‐retention capacity as a result of interactions between the phenolic compounds in HLPE and gluten proteins. Fibrous structures or high polyphenol content in the hawthorn leaf extract may have disrupted the continuity of the gluten network, preventing the bread from expanding sufficiently during baking. Furthermore, the combination of increased weight (due to higher water‐holding capacity) and decreased volume is the primary reason for the lower specific volume compared to the control group. This indicates that HLPE addition leads to a denser and less porous bread structure. Comparable results have been reported in the literature. In a study by Tolve et al. (2021), grape pomace powder, a by‐product of wine production, was incorporated into bread formulations as a flour substitute at levels of 0%, 5%, and 10%. The authors reported a progressive decrease in the specific volume of bread samples with increasing grape pomace powder content. The specific volume values of breads produced with 0%, 5%, and 10% grape pomace powder were reported as 5.52, 3.59, and 2.82 cm^3^/g, respectively. These findings are consistent with the present results.

The moisture content of the bread samples was found to range between 24.97% and 26.64%, and HLPE addition had a statistically significant effect on moisture levels (*p* < 0.05). While the highest moisture content was detected in the sample containing 1.20% HLPE, the lowest moisture value was recorded in the 0.90% HLPE group. The differences between the control group and the samples supplemented with 0.30%, 0.60%, and 1.50% HLPE were found to be statistically insignificant (*p* > 0.05).

The fluctuations observed in moisture content up to the 1.20% level, and the peak at this concentration, can be explained by the hydrophilic nature of the hawthorn leaf phenolic extract. Phenolic compounds and potential polysaccharide residues in the extract can form hydrogen bonds with water molecules, making it more difficult for water to evaporate during baking (Sivam et al. 2010; Girard and Awika 2020). The fact that moisture reached its highest level at the 1.20% dosage suggests that this concentration provides an optimum interaction for water‐holding capacity. However, the subsequent decrease at the 1.50% level might be due to excessive phenolics disrupting the gluten structure, thereby hindering the entrapment of water within the matrix.

Upon examining the ash content of the bread samples, values were found to range between 1.87% and 2.05%. It was determined that the addition of HLPE did not create a statistically significant difference in the ash content of the breads (*p* > 0.05). The control group and all HLPE‐supplemented samples were classified within the same statistical group. Although a numerically small downward trend was observed as the HLPE concentration increased, this did not constitute a significant difference among the samples. Ash content is an indicator of the total mineral matter in foods (AOAC 2005). The lack of a significant increase in ash values despite HLPE addition indicates that the mineral contribution of the hawthorn leaf extract is negligible compared to the primary bread ingredients. Furthermore, due to the extraction method being phenolic‐focused, the transfer of mineral substances into the extract might have remained limited. These results are consistent with studies in the literature suggesting that the use of plant extracts at low rates (generally < 2%) does not majorly affect the total ash content of bakery products (Dziki et al. 2014).

The hardness values of the bread samples ranged from 6.21 N to 11.90 N, and the addition of HLPE was found to cause a statistically highly significant increase in bread texture (*p* < 0.05). While the lowest hardness value was measured in the control group, it was observed that every increase in HLPE concentration significantly raised the hardness value. When the HLPE ratio reached 1.50%, the hardness of the bread approximately doubled compared to the control group, reaching 11.90 N. The fact that each sample group is statistically distinct demonstrates the potent effect of the HLPE dosage on textural hardness. This steady increase in hardness values is directly correlated with the decrease in specific volume (Dziki et al. 2014). Breads with reduced volume and a more compact structure exhibit a firmer internal crumb texture. The cross‐linking of phenolic compounds with gluten proteins may have reduced the elasticity of the gluten network, leading to the formation of a stiffer and more resistant matrix (Girard and Awika 2020). Additionally, the high water‐holding capacity of HLPE may have altered the interaction of water with flour components (starch and protein) within the dough, triggering a denser structure after baking (Sivam et al. 2010). Findings in the literature suggesting that plant extracts and polyphenols increase hardness while decreasing bread volume support these results (Świeca et al. 2014).

### 3.2 Crumb and Crust Color Parameters of Bread Samples

The results of color values of the control bread and HLPE‐fortified bread samples at different concentrations (0.30%, 0.60%, 0.90%, 1.20%, and 1.50%) are presented in Table 2. The L* values, which indicate crust lightness, ranged from 49.99 to 67.73. The highest L* value for the crust was observed in the control bread, while a progressive decrease in L* values was recorded as the HLPE level increased. The lowest L* value was detected in the bread containing 1.50% HLPE, which was statistically different from all other samples. These results indicate a pronounced darkening of the bread crust with HLPE addition. The changes in crust color are attributed to the high‐purity phenolic content and chlorophyll pigments of the added extract. Since the extract is poor in reducing sugars and free amino acids, the observed darkening (decrease in L*) is likely due to the thermal oxidation of phenolic compounds into dark‐colored polymeric structures (such as quinones) at baking temperatures, while Maillard reaction products may also contribute to crust browning during baking, as commonly reported in thermally processed cereal‐based systems (Sivam et al. 2010; Nayak et al. 2015).

**TABLE 2:** Color properties of control bread and HLPE‐fortified bread samples at different concentrations (0.30%–1.50%).

Bread Samples	L*	a*	b*	C*	h°
Crust					
Control	67.73 ± 0.70^a^ **	11.07 ± 0.98^a^	29.62 ± 0.37^ab^	31.63 ± 0.53^ab^	69.51 ± 1.64^b^
0.30% HLPE	63.70 ± 1.91^b^	9.27 ± 0.46^b^	30.38 ± 0.74^a^	31.76 ± 0.76^a^	73.02 ± 0.78^a^
0.60% HLPE	62.16 ± 2.40^b^	8.26 ± 0.83^bc^	29.06 ± 0.87^bc^	30.22 ± 0.81^cd^	74.12 ± 1.68^a^
0.90% HLPE	58.33 ± 3.13^c^	8.35 ± 0.33^bc^	29.30 ± 0.58^abc^	30.47 ± 0.56^bc^	74.09 ± 0.67^a^
1.20% HLPE	56.59 ± 1.01^c^	7.91 ± 0.79^c^	28.05 ± 0.71^c^	29.15 ± 0.51^d^	74.24 ± 1.83^a^
1.50% HLPE	49.99 ± 2.13^d^	7.75 ± 0.61^c^	28.15 ± 1.21^c^	29.20 ± 1.30^d^	74.62 ± 0.81^a^
Crumb					
Control	75.22 ± 1.81^a^	−0.21 ± 0.02^a^	14.60 ± 0.56^e^	14.60 ± 0.56^e^	90.82 ± 0.11^f^
0.30% HLPE	70.60 ± 0.66^b^	−0.36 ± 0.03^b^	16.54 ± 0.48^d^	16.54 ± 0.48^d^	91.26 ± 0.11^e^
0.60% HLPE	68.38 ± 1.38^c^	−0.87 ± 0.05^c^	17.53 ± 0.17^c^	17.55 ± 0.17^c^	92.82 ± 0.16^d^
0.90% HLPE	65.05 ± 0.52^d^	−1.15 ± 0.07^d^	18.49 ± 0.29^b^	18.53 ± 0.29^b^	93.55 ± 0.26^c^
1.20% HLPE	63.75 ± 1.09^de^	−1.39 ± 0.14^e^	19.26 ± 0.17^a^	19.31 ± 0.16^a^	94.14 ± 0.43^b^
1.50% HLPE	62.61 ± 0.81^e^	−1.75 ± 0.12^f^	19.60 ± 0.30^a^	19.67 ± 0.30^a^	95.11 ± 0.41^a^

*Note:* Results are given as mean ± standard deviation.

Abbreviation: HLPE, Hawthorn leaf phenolic extract.

** Small letters in the same column show difference between bread samples (*p* < 0.05).

The a* values of the crust ranged from 7.75 to 11.07, with the highest value exhibited by the control sample. A general decreasing trend in a* values was observed with increasing HLPE levels, particularly in breads containing 1.20% and 1.50% HLPE. The decrease in a* value reflects the green pigment effect of chlorophyll derived from the hawthorn leaves.

Significant differences were also observed in b* values among the samples. The highest b* value was recorded in the bread containing 0.30% HLPE, whereas higher HLPE concentrations (1.20% and 1.50%) resulted in reduced b* values.

Regarding C*, a significant decrease was observed in breads containing higher HLPE levels, especially at 1.20% and 1.50%, indicating reduced color saturation. Conversely, h° values increased with HLPE addition, and the control bread was statistically different from all HLPE‐enriched samples. The h° value reflects the green pigment effect of chlorophyll derived from the hawthorn leaves. The presence of green pigments suppressed the natural reddish‐brown tones of the bread crust, shifting the color spectrum toward the yellow‐green axis. Furthermore, potential changes in dough pH caused by the phenolic extract may have influenced the stability of existing pigments, contributing to a duller and darker crust appearance. Overall, crust color development appears to be governed by a combination of pigment incorporation, phenolic oxidation, and thermally induced browning reactions.

The L* values of the crumbs ranged from 62.61 to 75.22. The highest lightness was observed in the control bread. A consistent and statistically significant decrease in L* values was observed with increasing HLPE levels. The lowest L* value was recorded in the bread containing 1.50% HLPE, indicating a darker crumb color compared to the control and low‐level HLPE samples. The changes observed in crumb color parameters can be primarily attributed to the green color and chlorophyll content of the hawthorn leaf phenolic extract. The direct incorporation of chlorophyll and its derivatives into the dough matrix likely contributed to the reduction in crumb lightness and the development of a darker crumb appearance.

The a* values of the bread samples' crumbs were all negative, ranging from −0.21 to −1.75, which indicates the presence of greenish tones. The control sample exhibited the highest (least negative) a* value, whereas a progressive decrease in a* values was observed with increasing HLPE concentration. Each increase in HLPE level resulted in a statistically significant difference in a* values compared to the other samples (*p* < 0.05). The increasingly negative a* values with higher HLPE addition suggest an enhancement of green color intensity in the crumb, which may be attributed to the chlorophyll content of hawthorn leaf phenolics. Despite thermal processing during baking, chlorophyll‐derived pigments appear to have contributed to the overall color profile of the crumb.

The b* values of the crumbs ranged between 14.60 and 19.60, increasing significantly with higher HLPE levels. The highest b* values were observed in breads containing 1.20% and 1.50% HLPE. A similar increasing trend was observed for C* and h° values. Unlike the crust, where Maillard reactions play a dominant role, the crumb undergoes limited non‐enzymatic browning. Therefore, the color changes observed in the crumb are mainly governed by the pigment composition and concentration of the extract, making the effect of HLPE more pronounced in the crumb than in the crust.

### 3.3 Total Phenolic Content and Total Flavonoid Content of Bread Samples

The TPC and TFC values of the bread samples are presented in Table 3. According to the analysis results, a statistically significant increase in the TPC and TFC values of the bread was observed as the rate of HLPE addition increased (*p* < 0.05). The TPC content, which was 64.77 mg GAE/100 g in the control group bread, increased approximately 4.2‐fold with the addition of 1.50% HLPE, reaching a value of 272.84 mg GAE/100 g. Similarly, while TFC values were 50.69 mg QE/100 g in the control group, they reached a level of 234.23 mg QE/100 g at the highest concentration (1.50%). A strong positive correlation was observed between TPC and TFC values (*r* = 0.98), indicating a close relationship between phenolic and flavonoid compounds in HLPE‐fortified bread samples. In addition, TPC showed a strong positive correlation with hardness (*r* = 0.96), suggesting that increased incorporation of HLPE phenolics contributed to a firmer crumb structure, likely due to interactions between phenolic compounds and gluten proteins, which may enhance matrix rigidity. Conversely, TPC was negatively correlated with loaf volume (*r* = −0.76) and specific volume (*r* = −0.79), indicating that higher phenolic levels may interfere with dough expansion and gas retention during fermentation, possibly through dilution of the gluten network and/or competitive interactions with starch–protein structures. Overall, these findings suggest that while HLPE enrichment improves the functional (phenolic) quality of bread, it simultaneously induces structural modifications that negatively affect bread‐making performance at higher supplementation levels (Table S1).

**TABLE 3:** TPC and TFC values of control bread and HLPE‐fortified bread samples at different concentrations (0.30%–1.50%).

Bread samples	TPC (mg GAE/100 g bread)	TFC (mg QE/100 g bread)
Control	64.77 ± 4.22^f^ *	50.69 ± 1.75^e^
0.30% HLPE	111.68 ± 3.47^e^	61.15 ± 2.01^e^
0.60% HLPE	169.01 ± 5.79^d^	111.31 ± 5.56^d^
0.90% HLPE	211.01 ± 7.64^c^	157.46 ± 9.46^c^
1.20% HLPE	238.10 ± 4.88^b^	192.69 ± 8.32^b^
1.50% HLPE	272.84 ± 6.07^a^	234.23 ± 12.92^a^

*Note:* Results are given as mean ± standard deviation.

Abbreviations: HLPE, Hawthorn leaf phenolic extract; TFC, Total flavonoid compound; TPC, Total phenolic compound.

* Small letters in the same column show difference between bread samples (*p* < 0.05).

Hawthorn leaves and extracts are known to contain considerable amounts of phenolic and flavonoid compounds such as vitexin, rutin, hyperoside, and other flavanol glycosides, which contribute to high total phenolic/flavonoid contents in plant extracts. The primary source of this dramatic increase in the breads is the secondary metabolites naturally present in hawthorn leaves and concentrated through extraction. Indeed, studies on *Crataegus* spp. demonstrate that phenolic acids and flavonoids are abundant in leaves and other plant parts, and the total phenolic and flavonoid contents are strong contributors to antioxidant activity (Alirezalu et al. 2018). The basal level of phenolic content detected in the control group originates from phenolic acids such as ferulic acid, which are naturally present in wheat flour (Wang et al. 2013). Previous research on cereal products enriched with plant extracts has shown that the incorporation of bioactive plant phenolics can significantly increase phenolic and antioxidant levels in bread (Świeca et al. 2014; Purkiewicz et al. 2024). The sharp increase, particularly in flavonoid content with the addition of HLPE, confirms that characteristic compounds of hawthorn leaves were successfully incorporated into the bread matrix. The fact that the amount of phenolic substances remained so high despite high‐temperature processes like baking suggests that hawthorn leaf phenolics have relatively high thermal stability or are protected against thermal degradation by forming complexes with starch and proteins within the bread matrix. Although processing can affect certain phenolic compounds, some flavonoids and glycosides from hawthorn extracts have been reported to persist after heat treatment. These results indicate that the produced bread can be categorized as functional foods.

### 3.4 Antioxidant Activity of Bread Samples

The antioxidant capacities of the breads enriched with HLPE were evaluated using ABTS, DPPH, and FRAP methods, and the results are presented in Table 4. The ABTS values ranged from 10.11 to 308.63 mg TE/100 g bread. The lowest ABTS value was observed in the control sample, while a consistent and statistically significant increase was recorded with increasing HLPE levels. The highest ABTS value was obtained in the bread containing 1.50% HLPE, which was significantly different from all other samples. Similarly, DPPH radical scavenging activity ranged between 83.27 and 162.47 mg TE/100 g bread and increased significantly in a dose‐dependent manner with HLPE addition. The FRAP reducing power values varied from 2.50 to 193.47 mg TE/100 g bread. A pronounced increase in FRAP values was observed with HLPE addition, particularly at HLPE levels of 0.60% and above. Strong positive correlations were found between TPC and antioxidant assays (ABTS: *r* = 0.96; DPPH: *r* = 0.95; FRAP: r = 0.96), as well as between TFC and antioxidant activities (ABTS: *r* = 0.99; DPPH: *r* = 0.97; FRAP: *r* = 0.98), indicating that phenolic and flavonoid compounds were the major contributors to the antioxidant capacity of HLPE‐fortified bread samples.

**TABLE 4:** Antioxidant activity of control bread and HLPE‐fortified bread samples at different concentrations (0.30%–1.50%).

Bread samples	ABTS (mg TE/100 g bread)	DPPH (mg TE/100 g bread)	FRAP (mg TE/100 g bread)
Control	10.11 ± 1.67^f^ *	83.27 ± 2.75^f^	2.50 ± 0.53^f^
0.30% HLPE	34.47 ± 4.36^e^	91.87 ± 1.51^e^	31.77 ± 0.59^e^
0.60% HLPE	95.56 ± 11.49^d^	99.67 ± 1.51^d^	56.70 ± 3.37^d^
0.90% HLPE	146.82 ± 10.00^c^	121.87 ± 4.66^c^	102.06 ± 3.44^c^
1.20% HLPE	221.00 ± 6.81^b^	142.87 ± 5.44^b^	164.84 ± 2.56^b^
1.50% HLPE	308.63 ± 9.53^a^	162.47 ± 5.72^a^	193.47 ± 3.30^a^

*Note:* Results are given as mean ± standard deviation.

Abbreviations: ABTS, Cation radical scavenging activity; DPPH, Free radical scavenging activity; FRAP, Ferric reducing antioxidant power; HLPE, Hawthorn leaf phenolic extract.

* Small letters in the same column show difference between bread samples (*p* < 0.05).

The observed increases in antioxidant activity with HLPE addition are closely associated with the corresponding increases in TPC and TFC values reported in the previous section. Phenolic compounds and flavonoids are well known for their ability to scavenge free radicals and reduce metal ions, thereby contributing to antioxidant activity (Hassanpour and Doroudi 2023).

Although ABTS, DPPH, and FRAP assays are based on different antioxidant mechanisms, the consistent trends observed across all three methods suggest that HLPE contributes to an overall enhancement of the antioxidant capacity of bread. The pronounced increase in FRAP values indicates a substantial improvement in the electron‐donating and ferric‐reducing capacity of HLPE‐enriched breads. In contrast, the relatively lower response observed in the DPPH assay may be related to the steric characteristics and reaction kinetics of the DPPH radical, which can limit its interaction with certain phenolic compounds, particularly those with complex structures (Prior et al. 2005). Moreover, ABTS and FRAP assays are known to respond to a wider range of hydrophilic and lipophilic antioxidants, whereas the DPPH assay may underestimate the antioxidant activity of some flavonoids due to solvent and structural compatibility issues (Floegel et al. 2011). Overall, these findings indicate that HLPE enhances the reducing capacity of bread, thereby improving its functional antioxidant profile. Furthermore, interactions between phenolic compounds and Maillard reaction products formed during baking may have contributed to the overall antioxidant capacity (Patrignani et al. 2025). This suggests that HLPE‐enriched breads benefit not only from the intrinsic antioxidant properties of hawthorn leaf phenolics but also from newly formed antioxidant compounds generated during thermal processing. Consequently, the use of HLPE has not only provided a nutritional supplement to the breads but has also transformed them into functional foods with protective potential against oxidative stress.

### 3.5 In Vitro Bioaccessibility of Phenolics, Flavonoids and Antioxidant Activity of Breads

**FIGURE 2:**
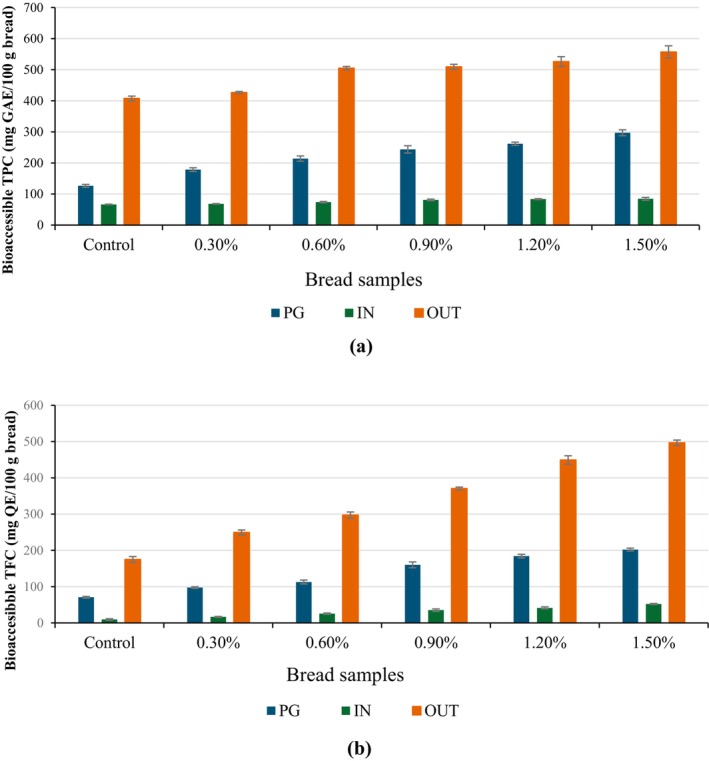
In vitro bioaccessibility of control and HLPE‐enriched breads (a) TPC values for digestion fractions, (b) TFC values for digestion fractions.

The change in TPC during the simulated gastrointestinal digestion phases (PG, IN, and OUT) is presented in Figure 2a. The TPC values in all digestive stages increased significantly as the HLPE fortification level increased (*p* < 0.05). In the PG phase, TPC values ranged from 126.31 to 297.06 mg GAE/100 g. The IN fraction, representing the bioaccessible portion, showed a lower range (66.48 to 84.49 mg GAE/100 g, corresponding to 14.03% and 13.17% bioaccessibility, respectively), with the %0.90, %1.20, and %1.50 groups showing statistically similar high recovery (*p* > 0.05). Remarkably, the highest TPC values were observed in the OUT fraction, reaching 557.04 mg GAE/100 g in the 1.50% HLPE sample, which is significantly higher than the gastric and dialyzable stages.

These results indicate that the simulated digestion process markedly influences the release and distribution of hawthorn leaf phenolics within the bread matrix. The higher TPC values observed in the PG phase compared to the undigested bread samples suggest that the acidic gastric environment, combined with pepsin activity, may partially disrupt the protein–starch network, thereby enhancing the extractability of phenolic compounds that were previously bound within the matrix (Parada and Aguilera 2007).

The IN fraction reflects the phenolic compounds that are potentially available for absorption in the small intestine rather than actual intestinal uptake. The comparatively lower TPC values in this phase may be associated with pH‐induced structural modifications, partial degradation, or interactions between phenolics and other food components under intestinal conditions, which can limit their diffusion across the dialysis membrane (Rodríguez‐Roque et al. 2013).

The markedly elevated TPC values in the OUT fraction suggest that a substantial proportion of phenolic compounds remain associated with insoluble components of the bread matrix, such as dietary fibers or protein aggregates, or forms high‐molecular‐weight complexes that are not dialyzable (Palafox‐Carlos et al. 2011). This behavior may also indicate strong interactions between phenolic compounds and the gluten–starch–fiber network formed during baking, which can limit their intestinal release. The predominance of phenolics in the OUT fraction indicates that a large share of the antioxidant constituents may reach the large intestine, where they could be subjected to microbial fermentation, potentially yielding smaller phenolic metabolites with biological relevance (Hervik and Svihus 2019). Such colonic biotransformation processes are known to enhance the bioactivity of bound phenolics by converting them into low‐molecular‐weight phenolic acids with improved absorption potential (Williamson and Clifford 2017). This behavior highlights the role of the bread matrix as a carrier system for delivering phenolic compounds to the lower gastrointestinal tract and supports the potential of HLPE‐fortified bread as a colonic‐targeted functional food system.

The changes in the TFC of the bread samples following simulated digestion are presented in Figure 2b. Statistical analysis revealed a significant increase in flavonoid levels across the PG, IN, and OUT phases relative to the HLPE supplementation rate (*p* < 0.05). In the PG phase, TFC values ranged from 70.68 to 202.14 mg QE/100 g. A notable reduction in flavonoid concentration was observed in the IN fraction, representing absorption from the small intestine (9.40 to 52.14 mg QE/100 g, corresponding to 5.10% and 9.50% bioaccessibility, respectively). Conversely, the highest flavonoid amount was recorded in the OUT phase, reaching 496.68 mg QE/100 g in the 1.50% HLPE‐fortified sample.

The reduction in TFC observed during the IN phase compared to the PG phase may be attributed to the sensitivity of certain flavonoids to intestinal conditions. Flavonoids commonly present in hawthorn leaves, such as quercetin derivatives and glycosides, are reported to be relatively stable under acidic gastric conditions but may undergo structural modification, degradation, or interaction with bile salts and digestive enzymes under mildly alkaline intestinal conditions (pH ≈7–7.5), potentially reducing their dialyzability (Manach et al. 2005; Rodríguez‐Roque et al. 2013). The higher flavonoid content detected in the OUT fraction compared to the IN fraction suggests that a considerable portion of flavonoids remains strongly associated with the bread matrix, likely through hydrogen bonding or hydrophobic interactions with dietary fibers and proteins. Progressive matrix degradation during digestion may enhance the extractability of these compounds rather than indicating their complete release or absorption. Consequently, a substantial amount of flavonoids may reach the colon, where they could serve as substrates for gut microbiota metabolism (Selma et al. 2009).

**FIGURE 3:**
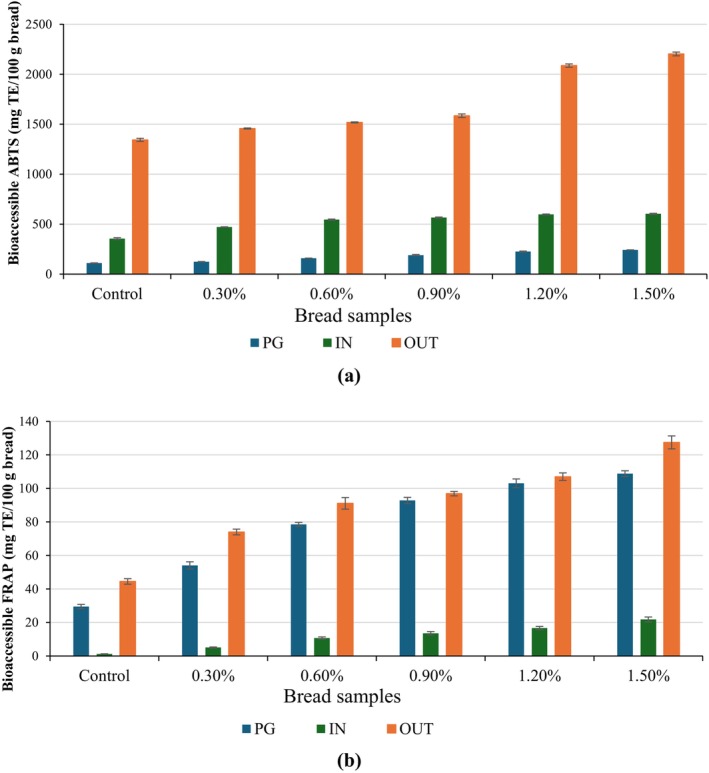
In vitro bioaccessibility of control and HLPE‐enriched breads (a) ABTS values for digestion fractions, (b) FRAP values for digestion fractions.

The ABTS radical scavenging activities of the bread samples throughout the simulated gastrointestinal digestion stages are shown in Figure 3a. A dose‐dependent increase in antioxidant capacity was observed in all digestion phases (PG, IN, and OUT) with increasing HLPE supplementation (*p* < 0.05). ABTS values in the PG phase ranged from 110.06 to 242.38 mg TE/100 g and increased in the IN fraction to values between 355.02 and 603.28 mg TE/100 g, corresponding to bioaccessibility values of 16.15% and 21.50%, respectively. The highest antioxidant activity was observed in the OUT fraction, reaching 2203.01 mg TE/100 g in the 1.50% HLPE‐fortified sample.

The higher ABTS activity observed in the IN phase compared to the PG phase suggests that qualitative changes in phenolic compounds may occur during digestion. In addition to deprotonation, conformational rearrangements, and enhanced solubility under intestinal conditions, digestive enzymes and changes in pH may disrupt the bread matrix, facilitating the release of phenolic compounds that were previously bound to macromolecules such as dietary fiber and proteins. Furthermore, partial hydrolysis of complex phenolic structures during digestion may generate lower‐molecular‐weight metabolites with enhanced radical scavenging capacity. These structural modifications may increase the accessibility of hydroxyl groups involved in electron or hydrogen atom transfer reactions, thereby enhancing antioxidant activity as measured by the ABTS assay. Moreover, because the ABTS assay detects both hydrophilic and lipophilic antioxidant compounds, whereas the FRAP assay primarily reflects the ferric reducing capacity of electron‐donating antioxidants, ABTS may be more sensitive to the diverse antioxidant species released during gastrointestinal digestion. Therefore, the increased ABTS activity observed after intestinal digestion likely reflects both improved extractability of antioxidant compounds and digestion‐induced structural modifications rather than the formation of entirely new antioxidant compounds (Su et al. 2019). The exceptionally high ABTS activity detected in the OUT fraction further supports the notion that a large proportion of antioxidant compounds remain bound to the bread matrix and is not dialyzable. This fraction may therefore contribute to antioxidant activity within the large intestine, potentially providing localized protection against oxidative processes in the colonic environment.

The FRAP values of the bread samples during the simulated gastrointestinal digestion phases are presented in Figure 3b. HLPE supplementation significantly increased the reducing capacity in all digestive fractions compared to the control (*p* < 0.05). In the PG phase, FRAP values showed a steady increase from 29.50 to 108.80 mg TE/100 g. Upon transition to the IN fraction, a substantial decrease was observed, with values ranging between 1.22 and 21.82 mg TE/100 g, corresponding to bioaccessibility values of 2.67% and 14.62%, respectively. However, the OUT fraction exhibited a recovery of activity, with the 1.50% HLPE sample reaching the highest value of 127.47 mg TE/100 g, surpassing both the PG and IN phases.

The pronounced decline in FRAP values in the IN phase may be related to the sensitivity of specific electron‐donating phenolic compounds to neutral or mildly alkaline conditions, as well as their potential interaction with proteins and polysaccharides, which can restrict their diffusion through the dialysis membrane (Wanyo et al. 2024). The higher reducing capacity observed in the OUT fraction indicates that a significant portion of redox‐active compounds remain within the non‐dialyzable digesta. Rather than being lost during digestion, these compounds may persist in the intestinal lumen, suggesting that the bread matrix effectively transports reducing agents through the gastrointestinal tract, where they may reach the large intestine and could undergo microbial transformation, potentially contributing to colonic antioxidant activity.

## 4 Conclusion

The present study suggests that HLPE can be successfully integrated into bread formulations, significantly enhancing phenolic content and antioxidant potential. Significant increases in TPC and TFC contents, as well as ABTS, DPPH, and FRAP antioxidant responses, were observed with increasing HLPE supplementation, indicating the potential of hawthorn leaves as a rich source of bioactive compounds. From a technological perspective, the addition of HLPE influenced the quality of the bread, particularly with regard to loaf volume, specific volume, texture, and color. Moderate enrichment levels (up to 1.20%) enabled the production of bread with acceptable physical properties; however, higher supplementation (1.50%) resulted in a denser structure and increased hardness. This indicates that HLPE concentration is a critical factor in balancing functional enhancement with technological quality. Simulated gastrointestinal digestion revealed that the presence of phenolic compounds in HLPE‐fortified breads is substantially affected by digestive conditions. While gastric digestion increased the extractability of phenolic compounds, the intestinal dialyzable fractions contained lower amounts, indicating limited bioaccessibility. By contrast, a significant proportion of phenolic compounds and flavonoids remained in the non‐dialyzable fraction, retaining substantial antioxidant activity. This suggests that HLPE‐derived compounds are largely associated with the bread matrix and may potentially reach the large intestine, where they could exert localized antioxidant effects or undergo microbial transformation. Overall, the findings suggest that HLPE‐enriched bread may represent a promising functional food capable of delivering phenolic compounds throughout digestion. The results emphasize the importance of considering technological performance and gastrointestinal behavior when developing phenolic‐enriched bakery products and provide a basis for further studies investigating in vivo bioavailability and physiological effects. In addition, future studies should also address sensory properties and consumer acceptance, as these factors are critical for the successful commercial application of functional bread formulations.

## Author Contributions

**Cemal Kaya:** writing – review and editing. **Esra Esin:** methodology, formal analysis. **Mustafa Bayram:** supervision, methodology, writing – review and editing. **Semra Topuz Türker:** conceptualization, software, investigation, methodology, formal analysis, writing – original draft, data curation.

## Funding

Open access funding provided by the Scientific and Technological Research Council of Türkiye (TÜBİTAK).

## Ethics Statement

The authors have nothing to report.

## Conflicts of Interest

The authors declare no conflicts of interest.

## Supporting information


**Table S1:** Pearson correlation matrix showing relationships among bioactive compounds, antioxidant activities, and technological quality of HLPE‐enriched bread formulations.

## Data Availability

The data that support the findings of this study are available from the corresponding author upon reasonable request.
