# Supplementary material for: Development of Functional Bread Fortified with Hawthorn Leaf Phenolic Extract: Evaluation of Technological Performance and Phenolic Bioaccessibility
Source: Food Sci Nutr. 2026 Aug 4;14(8):e72217. doi: 10.1002/fsn3.72217 (PMC13437948; doi:10.1002/fsn3.72217)
Supplement: Supplementary file 1 [file FSN3-14-e72217-s001.docx]

Table S1. Pearson correlation matrix showing relationships among bioactive compounds, antioxidant activities, and technological quality of HLPE-enriched bread formulations

| **Pearson correlation coefficients (r)** | **TPC** | **TFC** | **ABTS** | **DPPH** | **FRAP** | **Hardness** | **Volume** | **Specific volume** |
| --- | --- | --- | --- | --- | --- | --- | --- | --- |
| **TPC** | 1.00 | 0.98 | 0.96 | 0.95 | 0.96 | 0.96 | -0,76 | -0.79 |
| **TFC** | 0.98 | 1.00 | 0.99 | 0.97 | 0.98 | 0.95 | -0.76 | -0.78 |
| **ABTS** | 0.96 | 0.99 | 1.00 | 0.99 | 0.99 | 0.97 | -0,79 | -0.82 |
| **DPPH** | 0.95 | 0.97 | 0.99 | 1.00 | 0.99 | 0.96 | -0.79 | -0.81 |
| **FRAP** | 0.96 | 0.98 | 0.99 | 0.99 | 1.00 | 0.97 | -0.76 | -0.80 |
| **Hardness** | 0.96 | 0.95 | 0.97 | 0.96 | 0.97 | 1.00 | -0.80 | -0.83 |
| **Volume** | -0.76 | -0.76 | -0.80 | -0.79 | -0.76 | -0.80 | 1.00 | 0.99 |
| **Specific volume** | -0.79 | -0.78 | -0.82 | -0.81 | -0.79 | -0.83 | 0.99 | 1.00 |
